# Supplementary material for: Evaluation of data processing pipelines on real-world electronic health records data for the purpose of measuring patient similarity
Source: PLoS One. 2023 Jun 15;18(6):e0287264. doi: 10.1371/journal.pone.0287264 (PMC10270623; doi:10.1371/journal.pone.0287264)
Supplement: S1 File — (ZIP) [file pone.0287264.s001.zip › Supporting Information/Table_S1.docx]

**Table S1: Features ranked by degree of influence on the resulting similarity according to each pipeline N=200. Bolded entries indicate numerical features. Number and direction of arrows indicate rank changes compared to N=20.**

| **Rank** | **MCA (% RV)** | **MCA/PCA (% RV)** | **MCA/PCA/PCA (% RV)** | **AE (% RV)** |
| --- | --- | --- | --- | --- |
| **1** | Anxiety (43.1) | **FEV_1_% pred (39.6)** | CRS (4.7) | Atopy (6.0) |
| **2** | Depression (43.5) | Depression (39.6) **↑↑↑** | Therapy (13.5) | IHD (8.5) |
| **3** | Diabetes (53.9) | Anxiety (39.7) **↑↑↑** | Atopy (25.8) | Smoking (19.2)**↑↑** |
| **4** | Heart failure (58.4) **↑** | **Age at index (40.4)** ↓↓ | Anxiety (42.1) **↑↑↑↑** | Therapy (19.6) |
| **5** | Smoking (59.3)↓ | **BMI (44.8) ↓↓** | Depression (43.1)**↑↑** | GERD (19.9)**↑** |
| **6** | IHD (63.3) | **Eosinophils (45.3)↓↓** | **FEV_1_%pred (48.7)↑↑** | Sex (20.5)↓↓↓↓ |
| **7** | **Age (66.1) ↑** | IHD (51.7) **↑** | **Age (49.1)↑↑↑** | Diabetes (23.5) |
| **8** | Sex (66.4)↓ | Smoking (54.7)**↓** | IHD (51.0)**↑↑↑** | Heart failure (39.6) |
| **9** | **BMI (69.7)** | Heart failure (54.9) | **BMI (54.4)↑↑** | Anxiety (40.2)**↑** |
| **10** | Hypertension (86.9) | CRS (56.4)**↑** | Smoking (54.4)↓x6 | Hypertension (47.0)↓ |
| **11** | GERD (89.9)**↑** | Atopy (66.7)**↑** | Heart failure (54.6)**↑↑** | Depression (50.2) |
| **12** | Atopy (93.5)↓ | Sex (68.3)**↓↓** | **Eosinophils (55.5)** | CRS (86.3)**↑** |
| **13** | CRS (93.7) **↑** | Therapy (72.6) | Diabetes (56.8)↓x8 | **BMI (87.4)**↓ |
| **14** | **FEV1 %pred (96.2)**↓ | Diabetes (78.7) | Sex (69.1) | **Age (90.7)↑** |
| **15** | Therapy (99.6) | GERD (85.8) | Hypertension (73.6) | **FEV_1_%pred (95.6)**↓ |
| **16** | **Eosinophils (103.5)** | Hypertension (93.3) | GERD (74.6) | **Eosinophils (107.2)** |
|  | | | | |
| **MRV categorical** | 71.0 | 63.5 | 46.9 | 47.6 |
| **MRV numeric** | 83.9 | 42.5 | 51.9 | 95.2 |
| **MVR overall** | 74.2 | 58.3 | 48.2 | 47.6 |
