# Supplementary material for: Evaluation of data processing pipelines on real-world electronic health records data for the purpose of measuring patient similarity
Source: PLoS One. 2023 Jun 15;18(6):e0287264. doi: 10.1371/journal.pone.0287264 (PMC10270623; doi:10.1371/journal.pone.0287264)
Supplement: S1 File — (ZIP) [file pone.0287264.s001.zip › Supporting Information/Table_S2.docx]

**Table S2: Features ranked by degree of influence on the resulting similarity according to each pipeline applied on the test dataset (N=20). Bolded entries indicate numerical features. Number and direction of arrows indicate rank changes compared to training dataset, N=20.**

| **Rank** | **MCA (% RV)** | **MCA/PCA (% RV)** | **MCA/PCA/PCA (% RV)** | **AE (% RV)** |
| --- | --- | --- | --- | --- |
| **1** | Anxiety (40.9) | **FEV_1_% pred (32.5)** | CRS (0.8) | Atopy (0.9) |
| **2** | Depression (44.2) | **Age at index (34.4)** | Therapy (8.9) | IHD (3.9) |
| **3** | Diabetes (47.9) | Anxiety (37.6) **↑↑↑** | Atopy (12.6) | Sex (10.1) |
| **4** | Heart failure (55.0)**↑** | **BMI (38.4)**↓ | Anxiety (39.1)**↑↑↑↑** | Therapy (10.3) |
| **5** | Smoking (56.5)↓ | Depression (38.6) | Depression (40.2)**↑** | Smoking (10.8) |
| **6** | IHD (62.8) | **Eosinophils (38.7)**↓↓ | **FEV_1_% pred (40.8)↑** | GERD (13.5) |
| **7** | Sex (64.7) | Smoking (48.5) | Smoking (41.6)↓↓↓ | Diabetes (16.0) |
| **8** | **Age (65.0)** | IHD (50.1) | **Age (42.7)↑** | Heart failure (23.0) |
| **9** | **BMI (68.1)** | Heart failure (50.6) | Diabetes (44.3)↓↓↓↓ | Hypertension (32.1) |
| **10** | Hypertension (81.8) | CRS (51.8)**↑** | IHD (45.8) | Anxiety (31.8) |
| **11** | GERD (87.1)**↑** | Sex (60.2)↓ | **BMI (47.7)** | Depression (38.0) |
| **12** | Atopy (90.2)↓ | Atopy (65.8) | **Eosinophils (48.5)** | **BMI (74.3)** |
| **13** | **FEV1 % pred (92.7)** | Therapy (68.7) | Heart failure (49.1) | **FEV_1_% pred (82.8)↑** |
| **14** | CRS (94.6) | Diabetes (75.8) | Sex (60.2) | **Age (84.1)↑** |
| **15** | Therapy (100.0) | GERD (78.0) | Hypertension (62.9) | **CRS (84.2)**↓↓ |
| **16** | **Eosinophils (101.5)** | Hypertension (91.5) | GERD (63.4) | Eosinophils (101.5) |
|  | | | | |
| **MRV categorical** | 68.0 | 59.8 | 39.1 | 22.9 |
| **MRV numeric** | 81.8 | 36.0 | 44.9 | 85.7 |
| **MVR overall** | 72.1 | 53.8 | 40.6 | 38.7 |
