# Supplementary figures and images for: Evaluation of data processing pipelines on real-world electronic health records data for the purpose of measuring patient similarity
Source: PLoS One. 2023 Jun 15;18(6):e0287264. doi: 10.1371/journal.pone.0287264 (PMC10270623; doi:10.1371/journal.pone.0287264)

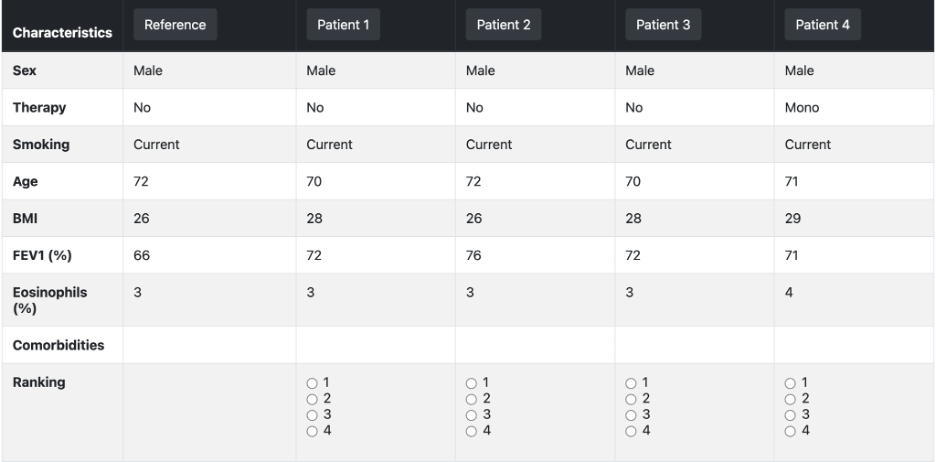

Supplement: S1 File — (ZIP) [file pone.0287264.s001.zip › Supporting Information/Figure_S1.tiff]
